# Global Metabolic Profiling of Infection by an Oncogenic Virus: KSHV Induces and Requires Lipogenesis for Survival of Latent Infection

**DOI:** 10.1371/journal.ppat.1002866

**Published:** 2012-08-16

**Authors:** Tracie Delgado, Erica L. Sanchez, Roman Camarda, Michael Lagunoff

**Affiliations:** 1 Department of Microbiology, University of Washington, Seattle, Washington, United States of America; 2 Molecular and Cellular Biology Program, University of Washington, Seattle, Washington, United States of America; University of Texas Southwestern Medical School, United States of America

## Abstract

Like cancer cells, virally infected cells have dramatically altered metabolic requirements. We analyzed global metabolic changes induced by latent infection with an oncogenic virus, Kaposi's Sarcoma-associated herpesvirus (KSHV). KSHV is the etiologic agent of Kaposi's Sarcoma (KS), the most common tumor of AIDS patients. Approximately one-third of the nearly 200 measured metabolites were altered following latent infection of endothelial cells by KSHV, including many metabolites of anabolic pathways common to most cancer cells. KSHV induced pathways that are commonly altered in cancer cells including glycolysis, the pentose phosphate pathway, amino acid production and fatty acid synthesis. Interestingly, over half of the detectable long chain fatty acids detected in our screen were significantly increased by latent KSHV infection. KSHV infection leads to the elevation of metabolites involved in the synthesis of fatty acids, not degradation from phospholipids, and leads to increased lipid droplet organelle formation in the infected cells. Fatty acid synthesis is required for the survival of latently infected endothelial cells, as inhibition of key enzymes in this pathway led to apoptosis of infected cells. Addition of palmitic acid to latently infected cells treated with a fatty acid synthesis inhibitor protected the cells from death indicating that the products of this pathway are essential. Our metabolomic analysis of KSHV-infected cells provides insight as to how oncogenic viruses can induce metabolic alterations common to cancer cells. Furthermore, this analysis raises the possibility that metabolic pathways may provide novel therapeutic targets for the inhibition of latent KSHV infection and ultimately KS tumors.

## Author Summary

In recent years there has been a resurgence in the study of metabolic changes in tumor cells. To determine if an oncogenic virus alters similar metabolic pathways as cancer cells, we measured the levels of a large number of metabolites in endothelial cells infected with Kaposi?s Sarcoma-associated herpesvirus (KSHV). KSHV is the etiologic agent of Kaposi's Sarcoma (KS), the most common tumor of AIDS patients world wide. Latent KSHV infection of endothelial cells altered a significant proportion of the host cell metabolites. Many metabolic pathways that are altered in most tumor cells were also altered by KSHV. In particular, KSHV upregulated fatty acid synthesis, a pathway that provides membrane material and metabolites critical for cell proliferation. Inhibitors of fatty acid synthesis kill many types of tumor cells and we found that these inhibitors led to death of cells latently infected with KSHV. In summary, we found that a directly oncogenic virus alters the same host metabolic pathways that are dysregulated in many cancer cells and that inhibition of these pathways can be used to kill off infected cells, thereby providing novel therapeutic targets for KSHV and ultimately KS tumors.

## Introduction

Many metabolic pathways are dramatically altered in cancer cells. These alterations are thought to provide cancer cells with the necessary energy and substrates for rapid cell division. Otto Warburg first demonstrated that most cancer cells have increased levels of glycolysis, even in the presence of oxygen, indicating that cancer cells dramatically alter their metabolism [1]. The increased aerobic glycolysis seen in most cancer cells, now termed the Warburg effect, is often accompanied by decreased oxygen usage, indicating a dramatic shift in the source of energy for tumor cells. Cancer cells become dependent on increased glycolysis and thus require increased glucose uptake for survival [2]–[5]. In addition to the Warburg effect, many other metabolic changes occur in most tumor cells, including increases in lipogenesis, amino acid metabolism, and the pentose phosphate pathway among others.

Recently, global changes in cellular metabolism have been studied using metabolomic approaches [6], [7]. Metabolomics generally involves the use of gas chromatography-mass spectrometry (GC-MS) and/or Liquid chromatography–mass spectrometry (LC-MS) to simultaneously detect changes in a wide variety of metabolites [6], [8]–[10]. Metabolomic approaches have allowed for the determination of global alterations of metabolism in tumor cells as well as in virally infected cells.

As non-living entities, viruses do not inherently have their own metabolism. However, upon infection, viruses dramatically alter the metabolism of the host cell. Viral alteration of host cell metabolism can provide the substrates necessary for viral replication. For example, alteration of host cell metabolism can provide the increased nucleotides necessary for genome replication or increased free amino acids needed for rapid viral protein synthesis. Virally-induced alterations of host metabolic pathways are likely to also be important for viral pathogenesis. Viral metabolomic studies were first used to identify changes in host cellular metabolism induced by human cytomegalovirus (HCMV) lytic infection [6], [7]. These studies found that HCMV infection leads to the alteration of many key metabolic pathways including changes that are essential for lytic replication. Subsequently, changes in the cellular metabolic profile were determined for cells infected by other viruses, including hepatitis C virus (HCV), human immunodeficiency virus (HIV) and herpes simplex virus (HSV) [10]–[12]. These studies identified numerous metabolic alterations in infected cells. However, metabolomic studies have yet to be done on viruses that directly induce oncogenesis.

Kaposi's Sarcoma-associated herpesvirus (KSHV) is the etiologic agent of Kaposi's Sarcoma (KS). KS is the most common tumor in parts of central Africa as well as in AIDS patients worldwide. KS lesions are predominantly populated by spindle cells of endothelial origin [13]. At late stages, all spindle cells in the tumor are infected with KSHV [14], [15]. Like all herpesviruses, KSHV has both lytic and latent viral phases. During lytic replication, there is broad viral gene expression and the virus is rapidly replicated, ultimately leading to cell death. During latency, viral gene expression is limited, supporting expression of genes necessary for the maintenance of the latent episome and for infected cell survival. In KS spindle cells, KSHV is predominantly in the latent state, however, a few percent of the cells express viral markers of lytic replication [16]. KSHV infection of cultured endothelial cells yields similar levels of latent and lytic infection [17].

Our previous studies found that KSHV infection of cultured endothelial cells directly induces the Warburg effect [18]. KSHV infection of endothelial cells leads to increased glucose uptake as well as increased lactic acid production. Expression of the glucose transporter, GLUT3, and the first rate-limiting enzyme in glycolysis, hexokinase-2, were also increased following latent infection. KSHV infection of endothelial cells also leads to significant decreases in oxygen consumption. Interestingly, both inhibition of lactate dehydrogenase, an essential enzyme for the conversion of pyruvate to lactate, or inhibition of glucose processing by a non-hydrolysable glucose analog, led to increased death of cells latently infected with KSHV [18]. These results indicate that glycolysis is required for the survival of cells latently infected with KSHV. This was the first evidence that KSHV directly induces a metabolic alteration common to most cancer cells. Furthermore, as observed in many cancer cells, this alteration is required for the survival of infected cells.

To determine if KSHV induces other metabolic changes similar to cancer cells, we performed a global screen to detect metabolites in mock- and KSHV-infected cells at 48 and 96 hours post infection (hpi). At 48 and 96 hpi, greater than 90% of the cells expressed a latent marker of infection, while only 1–5% of the cells expressed a marker of lytic replication. By 48 hpi, KSHV altered greater than one-quarter of the approximately 200 detectable metabolites and nearly one-third were altered by 96 hpi. Metabolites of several pathways commonly dysregulated in tumor cells were altered by KSHV, including glycolysis, amino acid metabolism, the pentose phosphate pathway and lipogenesis. KSHV infection leads to elevated levels of over half of the detectable metabolite products of *de novo* fatty acid synthesis (FAS or lipogenesis), and was concurrent with increased lipid droplets in latently infected endothelial cells. Lipid droplets are lipid-rich cytoplasmic organelles (diameter <1–100 µm) that store cellular energy in the form of triacylglycerols and also store phospholipids and sterols, the building blocks of membranes [19], [20]. Therefore, lipid droplets provide both readily accessible energy as well as cellular membrane material.

Fatty acids are essential substrates for energy metabolism and are the major components of all biological lipid membranes [21]. Animal fatty acids can be derived exogenously from diet or endogenously by *de novo* lipogenesis. Normal cells predominantly acquire fatty acids from dietary sources rather than *de novo* lipogenesis. However, the majority of fatty acids from cancer cells are derived by *de novo* lipogenesis even when exogenous fatty acids are available [22]. Induced lipogenesis is thought to aid in rapid cell division by generating the raw material needed for membrane production and the required metabolites for cell proliferation. Previous studies have shown that increased lipogenesis is required for the survival of many cancer cells [23]. We show here that the induction of FAS is also required for the survival of endothelial cells latently infected with KSHV. Inhibitors of FAS led to greatly increased apoptotic death of latently infected cells, as compared to their mock-infected counterparts. This effect was reversed by the addition of palmitc acid, the first fatty acid metabolite that is produced downstream of the drugs we used to block FAS. Therefore, the products of FAS are necessary for the survival of endothelial cells latently infected with KSHV. Inhibition of fatty acid synthesis provides a potential therapeutic target for KSHV and ultimately KS tumors.

## Results

### KSHV infection induces host cell metabolism

To determine global changes in host cell metabolism induced by latent KSHV infection, we utilized a metabolomic approach. Tert-immortalized human dermal microvascular endothelial cells (TIME cells) [17] were mock- or KSHV-infected and harvested at 48 and 96 hpi for metabolic profiling. As determined by immunofluorescence in all experiments, greater than 90% of the infected cells expressed the latency-associated nuclear antigen (LANA), a viral marker of latent infection, while less than 5% of the cells had detectable ORF 59 staining, a viral marker of lytic infection [17]. We harvested and analyzed metabolites from the lysates of duplicate infections at both 48 and 96 hpi in three independent experiments. Therefore, a total of six biological replicates at both time points were examined. Both GC-MS and LC-MS analysis was used to analyze a broad spectrum of metabolites and was performed by Metabolon (North Carolina). Detection of 194 distinct characterized biochemicals could be unambiguously identified in our samples. A large fraction of these metabolites were significantly altered by latent KSHV infection, nearing one-third of compounds by 96 hpi (TableS1). Specifically, at 48 hpi, 58 metabolites were determined to be altered with a p-value of <0.05, 45 metabolites were induced and 13 metabolites were decreased. By 96 hpi, 63 metabolites were determined to be altered with a p value of <0.05, 51 metabolites were induced and 12 metabolites were decreased.

A variety of curation procedures were carried out by Metabolon to ensure the quality of the data set presented. System artifacts, mis-assignments, and background noise were limited by Metabolon data analysts through the use of proprietary visualization and interpretation software to confirm the consistency of peak identification among the various samples. Bradford assays were used to normalize each sample by protein concentration and each biochemical in the mock samples was re-scaled to yield a median equal to 1. KSHV-infected cells are shown as fold change compared to mock-infected cells. 194 named biochemical were unambiguously identified in our samples. Following log transformation and imputation with minimum observed values for each compound, Welch's two–sample t-tests were used to determine which biochemicals are significantly altered by KSHV infection at 48 and 96 hpi across the 6 biological replicates. An estimate of the false discovery rate (q-value) was calculated to take into account the multiple comparisons that normally occur in metabolomic-based studies. Low q-values were calculated for significantly different biochemical (p≤0.05) for both comparisons, indicating a low estimated false discovery rate. The q-values for the significantly altered compounds are 0.06 and 0.09 at 48 hours and 96 hpi respectively.

Several major metabolic pathways that are often altered in cancer cells were significantly altered by KSHV infection (Table S1). In the KSHV latently infected cells there is a general increase in amino acid metabolism including a large number of amino acids and a number of metabolites involved in the anabolic pathways of these amino acids. Induction of amino acid metabolism is thought to allow for the increased biosynthetic demand of rapidly dividing cells. Polyamine metabolism intermediates putrescine, spermidine, and spermine were also found at elevated levels in KSHV infected samples at 48 and 96 hpi. Polyamines are important for eukaryotic cell growth and differentiation and increased polyamine synthesis has been associated with cancer cells. Supporting our previous findings that increased glucose uptake is required for the survival of KSHV infected cells [18], the metabolomic data show a significant increase in glucose in the infected samples at 96 hpi. The data also reveal increased levels of the major glycolytic intermediates 3-phosphoglycerate and 2-phosphoglycerate, and a significant increase in phosphoenolpyruvate (PEP). In addition to glycolysis, the metabolomic data indicate that glucose may also be utilized in the pentose phosphate pathway, which synthesizes both nucleotides and NADPH. The pentose phosphate pathway intermediates ribose 5-phosphate and the ribulose 5-phosphate and/or xylulose 5-phosphate (isobars that cannot be differentiated) were found at significantly elevated levels in KSHV infected samples at 48 hpi. Additionally, by 96 hpi, 6-phosphogluconate was measured at significantly elevated levels in KSHV infected samples. The elevation of these intermediates suggests that increased levels of nucleotides, nucleic acids and NADPH are required during infection. Importantly, it is known that NADPH is a necessary co-factor for fatty acid biosynthesis. In the KSHV-infected cells there were significant increases in many metabolites involved in *de novo* fatty acid synthesis (FAS) as described more extensively below. Of note, many of the metabolites that are increased by infection, are increased to higher levels at 96 hours as compared to 48 hours post infection. For example, increased glucose is only increased 1.38 fold at 48 hours with a p-value of greater than 0.05, while it is increased more than 10 fold at 96 hours with a p-value below 0.05 (Table S1). At 96 hpi, many of the metabolites involved in FAS are also increased to a greater extent than at 48 hpi, suggesting that most of the changes are unlikely due to binding and entry but rather are dependent upon KSHV gene expression.

### KSHV infection induces host cell lipogenesis

**Figure 1:**
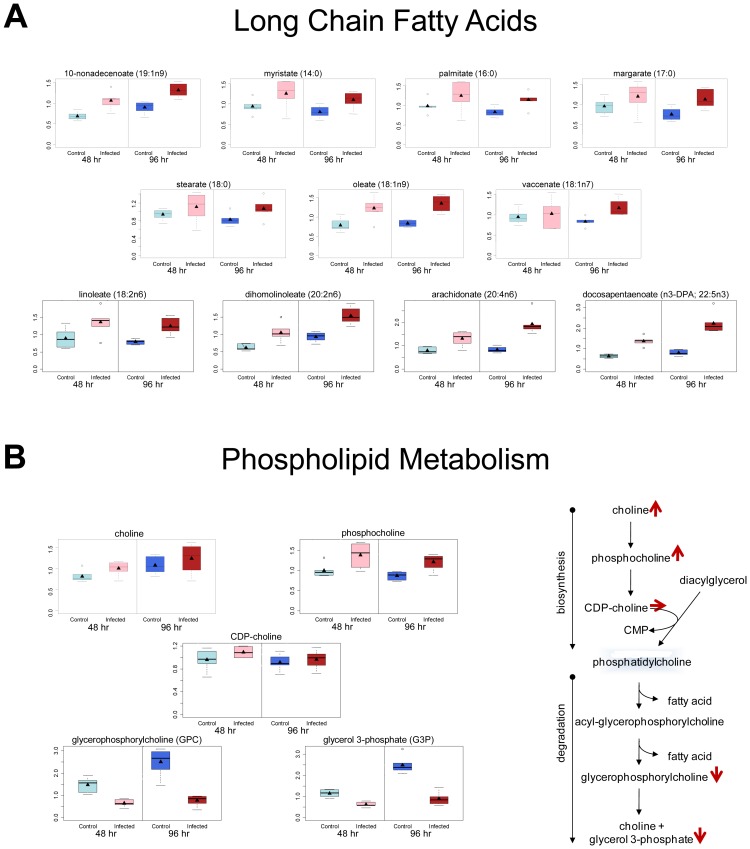
KSHV infection of endothelial cells induces fatty acid production through *de novo* synthesis. Relative levels of metabolites from mock- (control) and KSHV-infected (infected) TIME cells are shown at 48 hpi (light blue and pink respectively) and 96 hpi (dark blue and red respectively). **A**) Box and whisker plots showing relative levels of fatty acid metabolites significantly altered by KSHV infection. **B**) Box and whisker plots of metabolites that differentiate production of long chain fatty acids (LCFAs) by synthesis or degradation of phospholipids indicating that increased fatty acid metabolites in KSHV infected cells come from increased synthesis.

Lipogenesis is induced in many cancer cells and involves the production of fatty acids and lipid material. Induction of lipogenesis allows increased cell proliferation and cancer cell survival. Our metabolomics study identified significant increases in many long chain fatty acids (LCFA) (fig. 1A), the building blocks of lipid material. In KSHV-infected cells, over 50% of the LCFA that could be detected were significantly increased, while no LCFA were decreased (Table S1 and fig. 1). Observed elevation in LCFA may be due to increased lipid synthesis or degradation of phospholipids [24]. Analysis of the metabolomics data indicated an increase in choline and phosphocholine, fatty acid precursor metabolites generally associated with increased FAS, and decreases in glycerophosphorylcholine and glycerol-3-phosphate, metabolites associated with fatty acid production due to degradation of phospholipids (fig. 1B). This supports the conclusion that the increase in LCFAs in KSHV-infected cells is due to synthesis and not degradation of fatty acid containing material.

### KSHV infection induces lipid droplet formation

**Figure 2:**
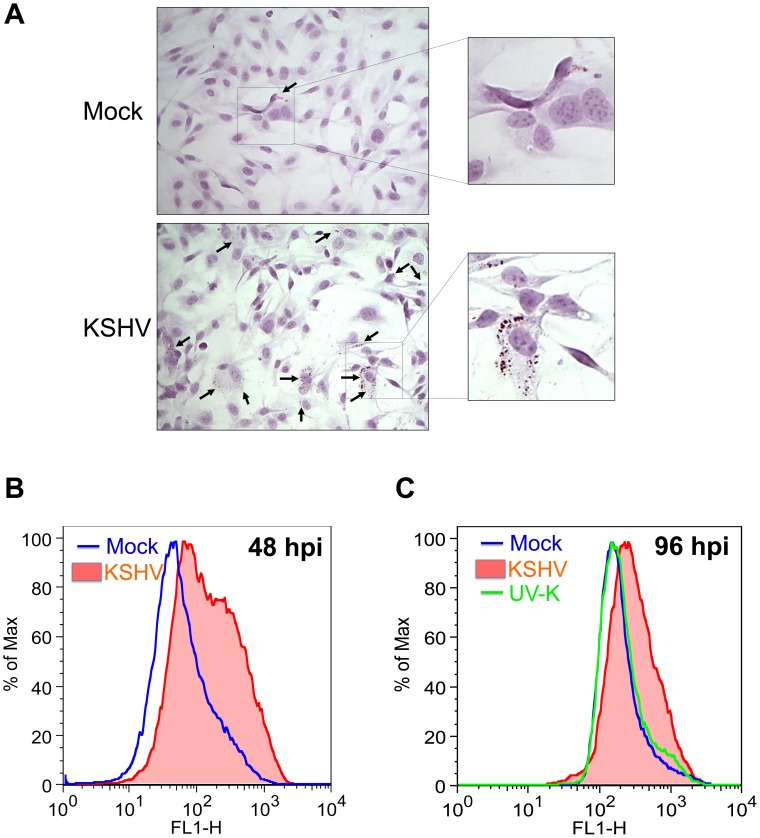
KSHV-infected cells induce the formation of lipid droplet organelles. **A**) 48 hpi, TIME cells were fixed and stained with Oil-Red-O, a specific stain for lipid droplets, and hematoxylin, to stain cell nuclei. Cells were visualized by bright field microscopy. **B**) Mock- and KSHV- infected TIME cells were harvested at 48 hpi, fixed and stained with Nile Red, a specific fluorescent stain for lipid droplets. Staining was analyzed by flow cytometry. **C**) Mock-, KSHV- and UV-irradiated-KSHV- infected TIME cells were harvested at 96 hpi and stained as in B for lipid droplets.

Lipid droplets are small cytoplasmic organelles that contain triacyglycerols for cellular energy storage, as well as, phospholipids and sterols for membrane production. Excess cellular LCFA can lead to the formation of lipid droplets [25]. To determine if increased lipogenesis in KSHV-infected cells leads to increased lipid droplet formation, mock- and KSHV-infected TIME cells were stained with Oil-Red-O, a specific stain for lipid droplets, and hematoxylin, a stain for cell nuclei. In every experiment greater than 85% of the cells expressed LANA, a marker of latent infection while less than 3% of the cells expressed ORF 59, a marker of lytic replication. Figure 2A shows representative images of high level staining for each condition. Many of the KSHV-infected cells had large numbers of lipid droplets while we only rarely saw significant lipid droplet staining in the mock-infected cells. To confirm and quantify this data we used flow cytometry to analyze mock- and KSHV-infected cells using another lipid droplet specific stain, Nile Red. There was much stronger Nile Red staining in KSHV-infected cells at 48 hpi as compared to mock-infected cells, indicating that, as was seen in the immunohistochemical staining, KSHV-infected cells have higher levels of lipid droplets (fig. 2B). To ensure that the increase in lipid droplets was due to viral gene expression we also stained cells infected with UV-inactivated KSHV. UV-inactivated virus still binds and enters cells, but viral gene expression is completely ablated. At 48 hpi, there was a slight increase in staining of the UV-inactivated virus as compared to mock infected cells but it was much lower than the levels seen in KSHV-infected cells (not shown). By 96 hpi, the staining of UV-inactivated virus was identical to mock-infected cells, while KSHV-infected cells still demonstrated a significant increase (fig. 2C). To determine if viral gene expression is continuously needed for lipid droplet formation or if lipid droplet formation is simply switched on by the virus, we passaged latently infected cells for over two weeks. As has been described before, long term passage of TIME cells leads to a loss of the viral episome [17]. At two weeks post infection,only around 40% of the cells maintain the viral episome with lower staining levels. Concurrent with loss of the viral episome, lipid droplet formation decreased to mock levels, indicating that continuous KSHV gene expression is required for lipid droplet formation (data not shown). Taken together, these data indicate that KSHV latent infection induces host cell lipogenesis and the infected cells store excess lipids in the form of lipid droplets.

### Fatty acid synthesis inhibitors induce apoptosis in KSHV-infected cells

**Figure 3:**
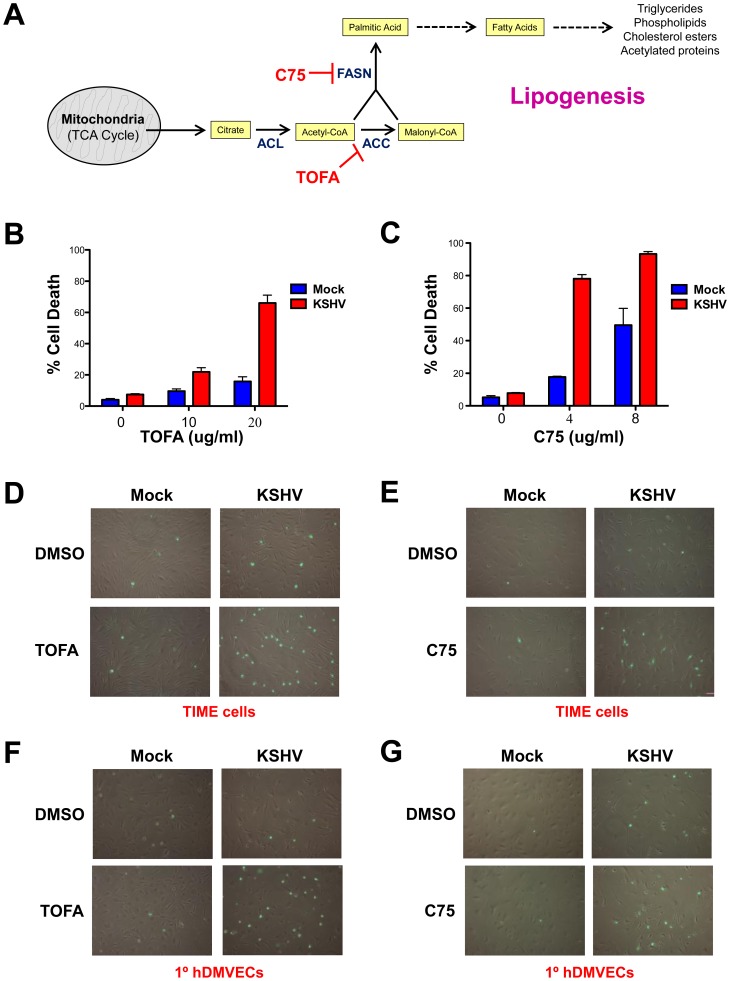
Fatty acid synthesis inhibitors selectively induce cell death in KSHV latently infected endothelial cells. **A**) Schematic of lipogenesis pathway and where specific inhibitors block the pathway. At 48 hpi, mock- and KSHV-infected TIME cells were treated with **B**) 0, 10, or 20 µg/mL of TOFA and incubated for an additional 48 hours or **C**) 0, 4 or 8 µg/mL C75 and incubated for an additional 24 hours. Cell death rates were then determined by a trypan blue exclusion assay. Cell death rate (%) is # of dead cells/# of total cells. 48 hpi, TIME cells (**D & E**) or 1° hDMVECs (**F & G**) were treated with 0 µg/mL or 20 µg/mL TOFA for 36 hours (C & E) or 0 µg/mL or 4 µg/mL C75 for 24 hours (D & F). Cells were then treated with Image-It Dead Green viability stain and visualized on an inverted fluorescent microscope.

Inhibitors of FAS have been shown to selectively kill cancer cells and inhibit tumor cell growth [23]. To determine if KSHV-induced lipogenesis is necessary for the maintenance of latently infected endothelial cells, we treated mock- and KSHV-infected TIME cells or 1°hDMVECs with the FAS inhibitors 5-(Tetradecyloxy)-2-Furoic Acid (TOFA) or C75 in three biological replicates. TOFA inhibits the enzyme, acetyl-CoA carboxylase (ACC1), thereby preventing the conversion of acetyl-CoA into malonyl-CoA during lipogenesis (fig. 3A).Tumor cells are very sensitive to TOFA treatment, usually leading to cancer cell death via apoptosis [26]. Forty-eight hpi, mock- and KSHV-infected TIME cells were treated with DMSO (control) or with TOFA for an additional 48 hours. After treatment, the percent of cell death was determined by a trypan blue exclusion assay. While KSHV-infected TIME cells have slightly higher basal cell death levels than their mock-infected counterparts, treatment with TOFA for 48 hours results in a very large dose-dependent increase in cell death in KSHV-infected cells while there is only a small increase in cell death in the mock-infected cultures (fig. 3B). After normalizing for the basal cell death rate in the absence of drug, around 60% of KSHV-infected cells die due to treatment with 20 µg/mL TOFA while only 9% of mock-infected cells die due to treatment with the same concentrations of TOFA.

Fatty acid inhibitor C75 specifically inhibits fatty acid synthase (FASN), thereby preventing the catalysis of acetyl-CoA and malonyl-CoA into palmitic acid (fig. 3A). Cancer cells are generally highly sensitive to C75 treatment compared to non-malignant cells. At 48 hpi, mock- and KSHV-infected cells were treated with C75 for 24 hours and cell death rates were determined by a trypan blue exclusion assay. Treatment with C75 for 24 hours induces a significantly larger dose-dependent increase in cell death in KSHV versus mock-infected cells (fig. 3C). After normalizing for the basal death rate in the absence of drug, 70% of KSHV-infected cells die due to 4 µg/mL C75 treatment, while only 13% of mock-infected cells die due to identical C75 treatment.

The FAS inhibitor results were confirmed in TIME cells (fig. 3D and E) and 1°hDMVECs (fig. 3F and G) by a microscopy-based fluorescence assay for dead cells (Image-It Dead Green Viability Stain). The fluorescent substrate is taken up by dead cells but is excluded from live cells. Treatment with either TOFA or C75 induced high levels of cellular fluorescence in KSHV-infected cells but not in mock-infected cells.

**Figure 4:**
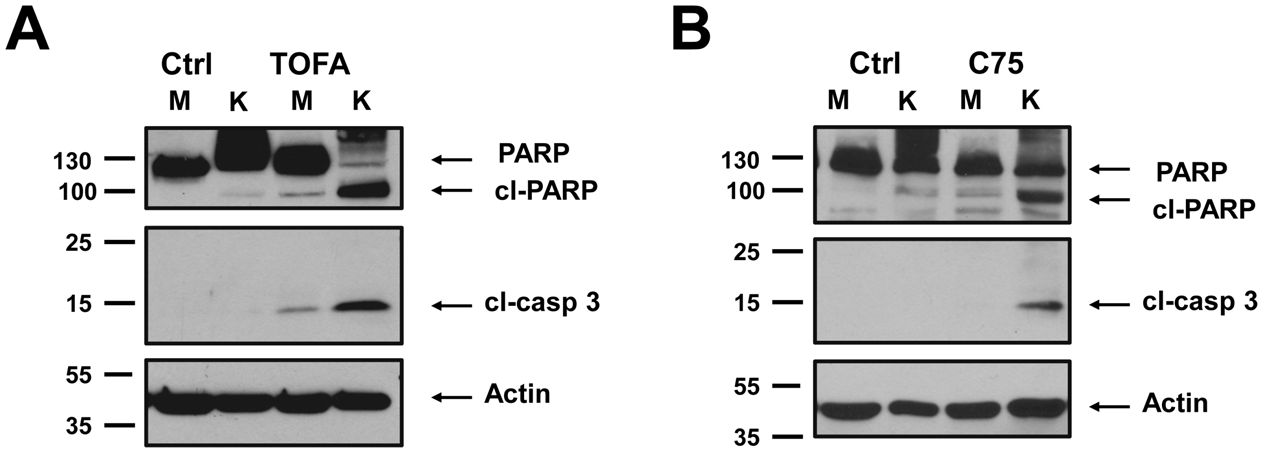
Fatty acid synthesis inhibitors selectively induce apoptosis in KSHV latently infected cells. 48 hpi, mock- (M) and KSHV- (K) infected cells were treated with **A**) 20 µg/mL TOFA for 36 hours or **B**) 4 µg/mL C75 for 24 hours and then harvested forWestern blot analysis with antibodies that recognize the cleaved PARP or cleaved caspase-3.

To determine the mechanism of cell death induced by TOFA and C75 in latently infected endothelial cells, classic markers of apoptosis were examined. Caspase 3 cleavage is an early event in many forms of programmed cell death and leads to the induction of PARP cleavage [27], [28]. Antibodies that recognize the cleaved forms of these proteins were used to determine if the treated cells are undergoing apoptosis. Endothelial cells were infected with KSHV for 48 hours and then treated with TOFA for an additional 36 hours or C75 for an additional 24 hours. Treatment of KSHV-infected cells with TOFA or C75 led to high levels of Caspase 3 and PARP cleavage (fig. 4A and B). Little or no induction of Caspase 3 or PARP cleavage was identified when mock-infected cells were treated with the same doses of C75 or TOFA. These results demonstrate that inhibition of fatty acid synthesis induces cell death by apoptosis specifically in KSHV-infected endothelial cells but not in mock-infected cells. Therefore, endothelial cells latently infected with KSHV require induced lipogenesis for survival.

### Metabolites downstream of palmitic acid are necessary for the survival of latently infected cells

**Figure 5:**
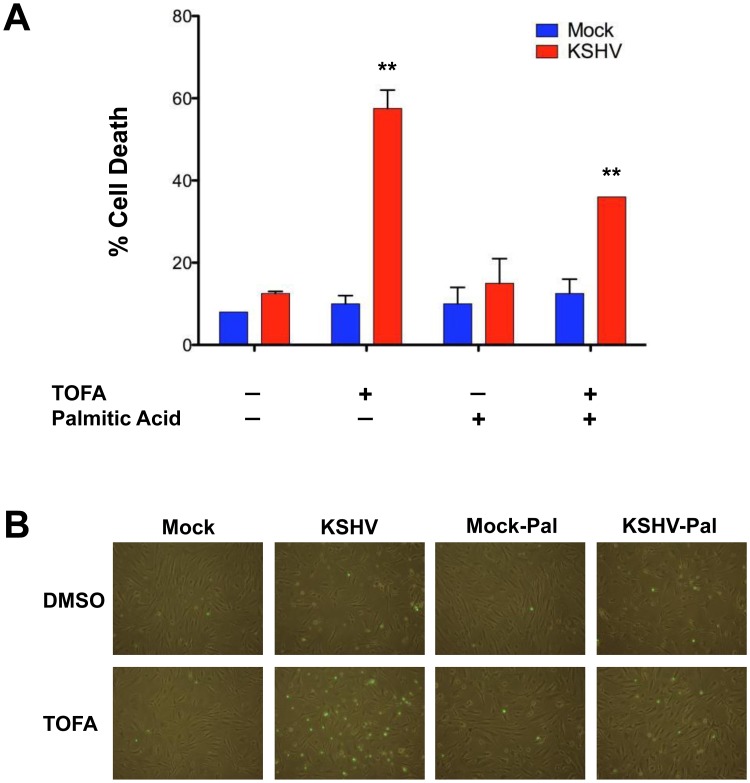
Palmitic Acid rescues TOFA induced cell death in KSHV latently infected endothelial cells. **A**) At 48 hpi, mock- and KSHV-infected TIME cells were treated with DMSO (control) or 20 µg/mL of TOFA, in the presence or absence of 24 µM Palmitic Acid, and incubated for an additional 48 hours. Cell death rates were then determined by a trypan blue exclusion assay. Cell death rate (%) is # of dead cells/# of total cells. **B**) At 48 hpi, TIME cells were treated as in panel A. Cells were then treated with Image-It Dead Green viability stain and visualized on an inverted fluorescent microscope.

Inhibition of ACC1 by TOFA could lead to apoptosis through inhibition of the synthesis of necessary downstream metabolites or through increased accumulation of precursors that could potentially be harmful to the cell. To determine if downstream pathways are necessary for latent infected cell survival we added palmitic acid, a metabolite that is downstream of the enzyme ACC1. Palmitic acid is the fundamental fatty acid precursor that iscreated by the conversion of acetyl-CoA and malonyl-CoA by the enzyme FASN. Addition of palmitic acid to tumor cell culture media has been shown to overcome blocks in fatty acid synthesis in tumor cells [29]. Therefore, we tested if the addition of palmitic acid could overcome TOFA induced cell death in KSHV infected endothelial cells. As seen above, 20 µg/ml of TOFA induces death in approximately 60% of the cells latently infected with KSHV but did not increase cell death in mock-infected cells (fig. 5A). Addition of palmitic acid slightly but not significantly increased cell death in untreated mock- and KSHV-infected cells and in mock-infected cells treated with TOFA (fig. 5A). However, the addition of palmitic acid significantly decreased the percentage of dead cells to around 35% in cultures of KSHV-infected cells treated with TOFA (fig. 5A). Thus, the addition of palmitic acid partially rescues KSHV infected cell death when a key step of fatty acid synthesis is blocked with TOFA. These results were confirmed with a fluorescent assay in TIME cells with a Dead Green viability stain (fig. 5B). These results indicate that KSHV-infectedcell death is not due to the build up oftoxic fatty acid synthesis precursors, butrather that the synthesis of downstream fatty acid metabolites is necessary for the survival of latent infection.

## Discussion

It has long been known that viruses can alter specific metabolic processes. However, global changes in metabolism have only recently been examined for a few lytic viruses. The first landmark study of viral metabolism was performed on HCMV infected cells [6]. This study found changes in many metabolic pathways including glycolysis and fatty acid synthesis. Subsequently, other viruses have been examined including HCV, HIV, Dengue virus and HSV [10]–[12], [30]. In all cases, there were dramatic changes in host cell metabolic pathways. Interestingly, viruses from different families induce some common metabolic pathways. For example, lytic infection with either HCMV or HCV induced glycolytic pathways [7], [10]. We recently demonstrated that KSHV also induces glycolysis during the latent phase of infection and this was confirmed by our metabolomics data [18]. We expected that the metabolic needs for latent and lytic phases of viral infection would be very different. However, the upregulation of glycolysis occurs both during latent KSHV infection and upon lytic infection with a slow growing herpesvirus, HCMV, but not with lytic infection by HSV, a rapidly replicating herpesvirus [7], [12], [18].

A number of enveloped lytic viruses have been shown to be inhibited by a block in FAS [7], [31]–[36]. It was proposed that lipogenesis provided membrane material for the envelopment of membrane containing viruses.Inhibition of FAS leads to decreased infectious virus for HCMV [7], influenza virus [7], Epstein-Barr virus (EBV) [31], varicella-zoster virus (VZV) [32], poliovirus [33], HCV [34], [35], yellow fever virus, West Nile virus and Dengue virus [36]. Interestingly, treatment of EBV infected cells with fatty acid synthase (FASN) inhibitors C75 and cerulenin lead to reduced immediate-early and early gene expression [31]. While the precise mechanism has yet to be investigated, it is not consistent with a block in envelopment. Dengue virus, which replicates on lipid droplets, has been shown to require induced fatty acid synthesis for replication at early stages as well [36]. Further work is necessary to determine if inhibition of FAS blocks KSHV lytic replication.

Interestingly, KSHV induces and requires lipogenesis during latent infection of endothelial cells. The increase in lipogenesis and lipid droplet accumulation was seen in the bulk of the population where greater than 85% of the cells expressed latent markers and less than 5% of the cells express lytic markers. This indicates that increased lipid droplets occurred in most of the latently infected cells. However, this does not rule out the possibility that lipid droplet formation requires a paracrine factor produced by lytic cells. Increased lipogenesis may predispose infected cells to oncogenesis as lipogenesis is upregulated in most cancer cells and is required for their survival. Inhibition of FAS led to high levels of apoptosis in KSHV latently infected cells, while the same concentration of inhibitors had little effect on the mock-infected cells. These effects were overcome by the addition of exogenous palmitic acid, a key FAS metabolite downstream of the early FAS inhibitor blockage. This indicates that downstream fatty acid metabolic processes are necessary for KSHV latency and that FAS inhibitors are not causing cell death simply due to build up of toxic pre-cursors. Importantly, apoptosis is also induced in many cancer cells following inhibition of FAS [23], [26], [37]. The question remains as to why KSHV latently infected cells require induced FAS. With regards to cancer cells, it has been hypothesized that induced FAS is necessary to meet the increased need for membranes due to high proliferation rates, as well as, for other important metabolic intermediates required for rapid cell division. However, in our hands, KSHV does not induce significant increases in proliferation of endothelial cells [38]. Therefore, KSHV likely requires specific FAS intermediates for survival or as a source of energy. Lipid droplet organelles can be used as energy storage for cells and could be critical for survival of the latently infected cells. Further experiments are necessary to identify the exact metabolites in the lipogenesis pathway that are necessary for the survival of cells latently infected with KSHV. The mechanism of induction of FAS by KSHV is also not clear. We did not see significant changes in the RNA levels or protein levels ofthree major FAS enzymes, ACC1, FASN or ACLY. We also found that induction of lipid droplets does not occur in all cell types as we did not see increased lipid droplets following high level infection of human foreskin fibroblast cells (data not shown). Further work on the cellular mechanisms are also ongoing.

All current drugs for herpesviruses specifically target only lytic replication steps. FAS inhibitors may provide novel targets for the inhibition of KSHV as they inhibit KSHV latency. Therefore, it is possible that FAS inhibitors could be used to treat KS tumors as they have the capacity to kill latently infected cells in the tumor. Overall, our metabolomics studies have identified a number of metabolic pathways altered by KSHV that are also commonly altered in cancer cells including glycolysis, amino acid synthesis, the pentose phosphate pathway, polyamine metabolism and fatty acid synthesis. These alterations in metabolism provide novel targets for inhibition of KSHV and possibly KS tumors. Experiments to identify the role of other metabolic pathways identified in our screen on KSHV pathogenesis are ongoing.

## Materials and Methods

### Cell lines

Tert-immortalized microvascular endothelial (TIME) cells or primary human dermal microvascular endothelial cells (1°hDMVECs) were maintained as monolayer cultures in EBM-2 media (Lonza or PromoCell) supplemented with a bullet kit containing 5%fetal bovine serum (FBS), vascular endothelial growth factor, basicfibroblast growth factor, insulin-like growth factor 3, epidermalgrowth factor, and hydrocortisone.

### Reagents and antibodies

Staurosporin (Sigma) was diluted in DMSO and were used at a final concentration of 1 µM. 5-(tetradecyloxy)-2-furancarboxylic acid (TOFA) (Sigma) and C75 (Calbiochem) were diluted in DMSO and used at the indicated final concentrations. Palmitic Acid (Sigma) was diluted in DMSO to 100 mM and used at a final concentration of 24 µM. Image-It Dead Green Viability Stain (Invitrogen) was diluted in DMSO and was used at a final concentration of 100 nM. Antibodies recognizing cleaved Caspase-3 (Cell Signaling), Poly-A Ribose Polymerase (Cell Signaling) ORF 59 (Advanced Biotechnology inc.) were used at 1∶1000 dilutions as specified by the manufacturer and antibodies to β-Actin (Sigma) was used at 1∶10,000. Antibodies that recognize KSHV protein, LANA (kind gift from Polson and Ganem) was used at 1∶1000 dilutions.

### Viruses, infection, and immunofluorescence

KSHV inoculum from induced BCBL-1 cells was concentrated, titered and used to infect TIME and primary hDMVECs as previously described [39]. All cells were infected in serum-free EBM-2 media for 3–4 hours, after which the medium was replaced withcomplete EGM-2. Infectionrates were assessed for each experiment by immunofluorescence and only experiments where greater than 85% of the cells expressed LANA and less than 5% of the cells expressed ORF59 were used. Prior to harvesting cells for immunoblot or enzymatic assays, an aliquotof mock- or KSHV-infected 1°hDMVECs or TIME cells were seeded on LabTekPermanoxfour-well chamber slides (Intermountain Sci) and fixed with4% (vol/vol) paraformaldehyde in phosphate-buffered saline. Infection rates were monitored using antibodies against thelatent KSHV protein LANA, as described previously [17]. For lytic replication, fixed cells were incubated with antibody to ORF 59 followed by incubation with fluor-conjugated secondary antibodies(goat anti-rabbit Alexa Fluor 488 and goat anti-mouse Alexa Fluor594 Molecular Probes/Invitrogen. Cells were mounted in medium containing DAPI (4′, 6′-diamidino-2-phenylindole)before being viewed under a Nikon Eclipse E400 fluorescencemicroscope (Nikon, Inc.).

### Immunoblot analysis

TIME cells were mock- or KSHV-infected, harvested using a cell scraper and pelleted. An aliquot of thecells was seeded onto chamber slides for immunofluorescenceanalysis as described above. Cell pellets were washed once in cold phosphate-buffered saline and then resuspended in RIPA lysis buffer (50 mMTris-HCl, pH 7.6, 150 mMNaCl, 1 mM EDTA, 1% NP-40, 0.5%deoxycholate, 0.1% sodium dodecyl sulfate, 1 mM sodium orthovanadate,1 mM sodium fluoride, 40 mM β-glycerophosphate, and CompleteMini protease inhibitor tablet [Roche]). Proteinconcentrations were determined by the bicinchoninic acid assay(Pierce). 15–50 µg of protein was fractionated on a sodiumdodecyl sulfate-polyacrylamide gel, transferred to a polyvinyldifluoride membrane, blotted with the indicated primary antibody diluted as described above, and subsequently with horseradish peroxidase-conjugated donkeyanti-goat (1∶20,000), goat anti-rabbit (1∶10,000), goat anti-mouse (1∶10,000).Immunoreactive proteins were visualized by chemiluminescence using the Amersham ECL Plus Western blotting detection reagents(GE Healthcare).

### Trypan blue exclusion assay and Image-It Dead Green Viability stain

48 hpi, mock- or KSHV-infected TIME cells or 1°hDMVECs were seeded sub-confluently (60,000 cells per well) into 12 well plates and treated with TOFA (0 µg/mL, 10 µg/mL or 20 µg/mL) for 48 hours or treated with C75 (0 µg/mL, 4 µg/mL or 8 µg/mL) for 24 hours. Cells were trypsinized and pelleted with cellular supernatant for 5 minutes and cell pellet was resuspended in ∼50 uL media. Cell death rates were determined by counting cells using a hemocytometer after addition of trypan blue, which is actively exported from the cytoplasm of live cells but remains in dead cells. Cell death rate (%) = number of dead cells/number of total cells (*100%). For the Dead Green viability assay, TIME cells or 1°hDMVECs were mock- or KSHV-infected for 48 hours, seeded sub-confluently in 12 well plates and treated 0 µg/mL or 4 µg/mL C75 for 24 hours, or 0 µg/mL or 20 µg/mL TOFA for 24 hours. Cells were then treated with Image-It Dead Green Viability stain for 15 min and pictures were taken with a fluorescent microscope. For Palmitic Acid rescue experiments, 48 hpi, mock- and KSHV infected TIME cells were seeded sub-confluently (60,000 cells/well) in 12 well plates and treated with TOFA at 20 µg/ml in the presence or absence of 24 µM Palmitic Acid for an additional 48 hours. Both trypan blue and Dead Green viability assays were completed as described above.

### Lipid droplet staining

48 hpi, mock- and KSHV-infected TIME cells were seeded in a chamber slide, fixed with 10% Formalin in PBS for 40–60 minutes and washed with 60% Isopropanol for 5 minutes. The cells were then exposed to fresh Oil-Red-O working solution for 10 minutes to stain the lipid droplets. Cells were then washed with dH2O, counterstained with hematoxylin for 1 minute to stain the nuclei and then washed and mounted in Aqua Poly/Mount (Polysciences Inc.). Pictures were taken under bright field microscopy. Images represent frames with high levels of lipid droplets for each condition.

### Flow cytometry

Mock- and KSHV-infected cells were trypsinized to remove cells from plates, counted and processed for flow cytometry. For detection of lipid droplets, the Lipid Droplets Florescence Assay Kit (Cayman Chemical) was used as recommended by the manufacturer. Briefly, 48 hpi TIME cells were washed in 1 mL Assay Buffer, fixed for 10 minutes at room temperature, washed with 1 mL Assay buffer, resuspended in Nile Red Staining Solution for 15 minutes at room temperature, washed and resuspended in Assay Buffer. Samples were analyzed by FACScan caliber flow cytometer (Becton-Dickinson, Franklin Lakes, NJ) or by FACS Canto flow cytometer. All Samples were analyzed by FloJo flow cytometry analysis software (Tree Star, Inc.; Ashland, OR).

## Supporting Information

Table S1 **Relative levels of allthe metabolites identified in KSHV infected cells compared to mock infected cells at 48 and 96 hours post infection.** Values shown are normalized levels from 6 distinct KSHV and mock infections of TIME cells. The major metabolic pathways are indicated in the right columns and the biochemical name and the mass spectrometry platforms used are indicated in the middle columns. Metabolites that are significantly increased (p<0.05) are shaded in red and those significantly decreased (p<0.05) are shaded in green. Numbers in blue are altered in KSHV infected cells but with lower statistical significance (0.05<p<0.01).
(DOCX)
