# Supplementary material for: Global Metabolic Profiling of Infection by an Oncogenic Virus: KSHV Induces and Requires Lipogenesis for Survival of Latent Infection
Source: PLoS Pathog. 2012 Aug 16;8(8):e1002866. doi: 10.1371/journal.ppat.1002866 (PMC3420960; doi:10.1371/journal.ppat.1002866)
Supplement: Table S1 — Relative levels of allthe metabolites identified in KSHV infected cells compared to mock infected cells at 48 and 96 hours post infection. Values shown are normalized levels from 6 distinct KSHV and mock infections of TIME cells. The major metabolic pathways are indicated in the right columns and the biochemical name and the mass spectrometry platforms used are indicated in the middle columns. Metabolites that are significantly increased (p<0.05) are shaded in red and those significantly decreased (p<0.05) are shaded in green. Numbers in blue are altered in KSHV infected cells but with lower statistical significance (0.05<p<0.01). (DOCX) [file ppat.1002866.s001.docx]

**TABLE S1-1:** **KSHV infection of endothelial cells alters global host cell metabolism.**

| **SUPER PATHWAY** | **SUB PATHWAY** | **BIOCHEMICAL NAME** | **PLATFORM** | **48hpi (KSHV vs Mock)** | **96hpi (KSHV vs Mock)** |
| --- | --- | --- | --- | --- | --- |
| Amino Acid | Glycine, serine and threonine metabolism | glycine | GC/MS | **1.37** | 1.08 |
|  |  | serine | GC/MS | **1.20** | 1.00 |
|  |  | N-acetylserine | GC/MS | 1.08 | **0.77** |
|  |  | threonine | GC/MS | 1.19 | 0.98 |
|  | Alanine and aspartate metabolism | alanine | GC/MS | **1.27** | 1.12 |
|  |  | beta-alanine | GC/MS | 1.21 | 0.98 |
|  |  | N-acetylalanine | LC/MS neg | 1.02 | 0.69 |
|  |  | aspartate | GC/MS | 0.81 | 0.75 |
|  |  | asparagine | GC/MS | **1.75** | **1.48** |
|  | Glutamate metabolism | glutamate | LC/MS pos | 1.10 | **1.18** |
|  |  | glutamine | LC/MS pos | **2.23** | **2.71** |
|  |  | pyroglutamine* | LC/MS pos | 0.99 | 1.07 |
|  |  | gamma-aminobutyrate (GABA) | GC/MS | 1.21 | 0.97 |
|  |  | N-acetylglutamate | LC/MS pos | **0.29** | **0.34** |
|  | Histidine | histidine | GC/MS | 1.07 | 0.92 |
|  | Lysine metabolism | cadaverine | GC/MS | **2.67** | 1.59 |
|  |  | lysine | GC/MS | **1.33** | 1.18 |
|  |  | 2-aminoadipate | GC/MS | 1.16 | 0.86 |
|  |  | N6-acetyllysine | LC/MS pos | 1.11 | 1.32 |
|  | Phenylalanine & tyrosine metabolism | phenylalanine | LC/MS pos | **1.14** | **1.13** |
|  |  | tyrosine | LC/MS pos | **1.15** | **1.21** |
|  | Tryptophan metabolism | kynurenine | LC/MS pos | **6.11** | **31.17** |
|  |  | tryptophan | LC/MS pos | 1.13 | 1.03 |
|  |  | C-glycosyltryptophan* | LC/MS pos | **1.35** | **1.55** |
|  | Valine, leucine and isoleucine metabolism | isoleucine | LC/MS pos | **1.21** | **1.17** |
|  |  | leucine | LC/MS pos | **1.29** | **1.21** |
|  |  | valine | LC/MS pos | **1.23** | **1.14** |
|  | Cysteine, methionine, SAM, taurine metabolism | cysteine | GC/MS | 1.14 | **0.55** |
|  |  | hypotaurine | GC/MS | 1.18 | 0.97 |
|  |  | S-adenosylhomocysteine | LC/MS neg | **1.41** | **1.34** |
|  |  | methionine | LC/MS pos | **1.23** | **1.20** |
|  |  | N-acetylmethionine | LC/MS neg | 1.08 | 0.93 |
|  |  | homocysteine | GC/MS | 0.75 | 0.73 |
|  | Urea cycle; arginine-, proline-, metabolism | dimethylarginine (SDMA + ADMA) | LC/MS pos | **1.74** | **1.78** |
|  |  | arginine | LC/MS pos | **1.34** | **1.53** |
|  |  | ornithine | GC/MS | **1.41** | **1.46** |
|  |  | proline | LC/MS pos | **1.29** | 1.18 |
|  |  | citrulline | LC/MS pos | **2.51** | **2.45** |
|  |  | trans-4-hydroxyproline | GC/MS | **1.68** | 1.04 |

Heat map of statistically significant biochemicals profiled in this study. For paired comparisons, shaded cells indicate p≤0.05 (red indicates that the mean values are significantly higher for that comparison; green values significantly lower). **Blue-bolded** text indicates 0.05< p< 0.10.

**TABLE S1-2:** **KSHV infection of endothelial cells alters global host cell metabolism.**

| **SUPER PATHWAY** | **SUB PATHWAY** | **BIOCHEMICAL NAME** | **PLATFORM** | **48hpi (KSHV vs Mock)** | **96hpi (KSHV vs Mock)** |
| --- | --- | --- | --- | --- | --- |
| Amino Acid | Creatine | creatine | LC/MS pos | 1.06 | 1.00 |
|  | Polyamine metabolism | 5-methylthioadenosine (MTA) | LC/MS pos | **0.71** | 0.90 |
|  |  | putrescine | GC/MS | **2.76** | **2.48** |
|  |  | agmatine | GC/MS | 1.53 | 1.33 |
|  |  | spermidine | LC/MS pos | **2.66** | **2.97** |
|  |  | spermine | GC/MS | **2.94** | **5.96** |
|  | Glutathione metabolism | glutathione, reduced (GSH) | LC/MS pos | **1.20** | 0.98 |
|  |  | 5-oxoproline | LC/MS neg | 1.15 | 1.10 |
|  |  | glutathione, oxidized (GSSG) | LC/MS pos | 1.21 | 1.19 |
|  |  | cysteine-glutathione disulfide | LC/MS pos | 1.25 | 1.06 |
| Peptide | Dipeptide | glycylglycine | GC/MS | **1.34** | 1.11 |
|  |  | glycylserine | GC/MS | 0.98 | 0.89 |
|  |  | glycylproline | LC/MS pos | 0.80 | 1.02 |
|  |  | glycylleucine | LC/MS pos | 1.09 | 1.19 |
|  |  | glycylglutamate | GC/MS | 1.05 | 0.90 |
|  |  | aspartylphenylalanine | LC/MS pos | 1.19 | 1.22 |
|  |  | cysteinylglycine | GC/MS | 0.89 | 0.73 |
|  | gamma-glutamyl | gamma-glutamylglutamate | LC/MS pos | 1.22 | 1.40 |
| Carbohydrate | Aminosugars metabolism | erythronate* | GC/MS | **0.64** | **0.62** |
|  |  | N-acetylneuraminate | GC/MS | 0.92 | 1.02 |
|  | Fructose, mannose, galactose, starch and sucrose metabolism | fructose | GC/MS | 1.06 | 1.13 |
|  |  | lactose | GC/MS | 0.85 | 0.65 |
|  |  | mannose-6-phosphate | GC/MS | 0.75 | **3.18** |
|  |  | sorbitol | GC/MS | **0.43** | **0.28** |

Heat map of statistically significant biochemicals profiled in this study. For paired comparisons, shaded cells indicate p≤0.05 (red indicates that the mean values are significantly higher for that comparison; green values significantly lower). **Blue-bolded** text indicates 0.05< p< 0.10.

**TABLE S1-3:** **KSHV infection of endothelial cells alters global host cell metabolism.**

| **SUPER PATHWAY** | **SUB PATHWAY** | **BIOCHEMICAL NAME** | **PLATFORM** | **48hpi (KSHV vs Mock)** | **96hpi (KSHV vs Mock)** |
| --- | --- | --- | --- | --- | --- |
| Carbohydrate | Glycolysis, gluconeogenesis, pyruvate metabolism | glycerate | GC/MS | **1.55** | **1.85** |
|  |  | glucose-6-phosphate (G6P) | GC/MS | 0.64 | 3.16 |
|  |  | glucose 1-phosphate | GC/MS | 1.20 | **0.33** |
|  |  | glucose | GC/MS | 1.38 | **10.53** |
|  |  | fructose-6-phosphate | GC/MS | 1.04 | 2.50 |
|  |  | fructose 1-phosphate | GC/MS | 1.59 | 0.93 |
|  |  | Isobar: fructose 1,6-diphosphate, glucose 1,6-diphosphate | LC/MS neg | 1.21 | 1.12 |
|  |  | 2-phosphoglycerate | GC/MS | 1.91 | 2.05 |
|  |  | 3-phosphoglycerate | GC/MS | 1.89 | **2.34** |
|  |  | dihydroxyacetone phosphate (DHAP) | GC/MS | 1.64 | 1.50 |
|  |  | phosphoenolpyruvate (PEP) | GC/MS | **2.90** | **4.31** |
|  |  | pyruvate | GC/MS | 1.59 | 0.98 |
|  |  | lactate | GC/MS | 1.18 | 0.75 |
|  | Nucleotide sugars, pentose metabolism | 6-phosphogluconate | LC/MS neg | 1.42 | **3.24** |
|  |  | ribitol | GC/MS | 0.79 | 0.73 |
|  |  | sedoheptulose-7-phosphate | GC/MS | 1.29 | 1.11 |
|  |  | gluconate | GC/MS | 0.57 | 1.04 |
|  |  | ribose | GC/MS | 1.19 | 1.04 |
|  |  | ribose 5-phosphate | GC/MS | **1.65** | 1.09 |
|  |  | Isobar: ribulose 5-phosphate, xylulose 5-phosphate | GC/MS | **1.79** | 1.39 |
|  |  | UDP-glucuronate | GC/MS | 1.05 | 1.16 |
| Energy | Krebs cycle | citrate | GC/MS | 1.00 | 1.12 |
|  |  | succinate | GC/MS | 1.00 | 0.86 |
|  |  | fumarate | GC/MS | **1.27** | **1.25** |
|  |  | malate | GC/MS | 0.97 | 0.93 |
|  | Oxidative phosphorylation | acetylphosphate | GC/MS | 1.12 | 1.28 |
|  |  | phosphate | GC/MS | 1.22 | 1.23 |
|  |  | pyrophosphate (PPi) | GC/MS | 1.48 | 1.17 |

Heat map of statistically significant biochemicals profiled in this study. For paired comparisons, shaded cells indicate p≤0.05 (red indicates that the mean values are significantly higher for that comparison; green values significantly lower). **Blue-bolded** text indicates 0.05< p< 0.10.

**TABLE S1-4:** **KSHV infection of endothelial cells alters global host cell metabolism.**

| **SUPER PATHWAY** | **SUB PATHWAY** | **BIOCHEMICAL NAME** | **PLATFORM** | **48hpi (KSHV vs Mock)** | **96hpi (KSHV vs Mock)** |
| --- | --- | --- | --- | --- | --- |
| Lipid | Essential fatty acid | dihomo-linolenate (20:3n3 or n6) | LC/MS neg | **1.99** | **3.14** |
|  |  | eicosapentaenoate (EPA; 20:5n3) | LC/MS neg | **1.68** | **2.63** |
|  |  | docosapentaenoate (n3 DPA; 22:5n3) | LC/MS neg | **2.15** | **2.80** |
|  |  | docosahexaenoate (DHA; 22:6n3) | LC/MS neg | **1.99** | **2.28** |
|  | Long chain fatty acid | myristate (14:0) | GC/MS | 1.34 | **1.37** |
|  |  | palmitate (16:0) | GC/MS | 1.27 | **1.38** |
|  |  | palmitoleate (16:1n7) | GC/MS | 0.98 | 1.12 |
|  |  | margarate (17:0) | GC/MS | 1.26 | **1.50** |
|  |  | 10-heptadecenoate (17:1n7) | GC/MS | 1.32 | 1.02 |
|  |  | stearate (18:0) | GC/MS | 1.18 | **1.31** |
|  |  | oleate (18:1n9) | LC/MS neg | **1.57** | **1.63** |
|  |  | cis-vaccenate (18:1n7) | GC/MS | 1.08 | **1.41** |
|  |  | linoleate (18:2n6) | LC/MS neg | **1.53** | **1.58** |
|  |  | 10-nonadecenoate (19:1n9) | LC/MS neg | **1.55** | **1.46** |
|  |  | arachidate (20:0) | GC/MS | 1.18 | **1.57** |
|  |  | eicosenoate (20:1n9 or 11) | LC/MS neg | **1.67** | **1.43** |
|  |  | dihomo-linoleate (20:2n6) | LC/MS neg | **1.70** | **1.65** |
|  |  | mead acid (20:3n9) | LC/MS neg | 1.38 | 1.23 |
|  |  | arachidonate (20:4n6) | LC/MS neg | **1.65** | **2.32** |
|  |  | behenate (22:0) | GC/MS | 1.14 | 1.27 |
|  |  | docosadienoate (22:2n6) | LC/MS neg | **1.65** | 1.22 |
|  |  | docosatrienoate (22:3n3) | LC/MS neg | **1.83** | **2.18** |
|  |  | adrenate (22:4n6) | LC/MS neg | 1.36 | **2.02** |
|  |  | lignocerate (24:0) | GC/MS | 1.29 | 0.99 |
|  |  | hexacosanoate | GC/MS | 0.69 | 0.72 |
|  | Fatty acid, ester | n-Butyl Oleate | GC/MS | 0.89 | 1.14 |
|  | Fatty acid, monohydroxy | 4-hydroxybutyrate (GHB) | GC/MS | 1.08 | **0.48** |
|  |  | 2-hydroxystearate | LC/MS neg | **1.77** | **3.01** |
|  |  | 2-hydroxypalmitate | LC/MS neg | **1.46** | **2.50** |
|  | Fatty acid, dicarboxylate | 2-hydroxyglutarate | GC/MS | 1.12 | 1.04 |
|  | Fatty acid, amide | oleamide | GC/MS | 1.69 | 1.71 |

Heat map of statistically significant biochemicals profiled in this study. For paired comparisons, shaded cells indicate p≤0.05 (red indicates that the mean values are significantly higher for that comparison; green values significantly lower). **Blue-bolded** text indicates 0.05< p< 0.10.

**TABLE S1-5:** **KSHV infection of endothelial cells alters global host cell metabolism.**

| **SUPER PATHWAY** | **SUB PATHWAY** | **BIOCHEMICAL NAME** | **PLATFORM** | **48hpi (KSHV vs Mock)** | **96hpi (KSHV vs Mock)** |
| --- | --- | --- | --- | --- | --- |
| Lipid | Glycerolipid metabolism | choline phosphate | LC/MS pos | **1.39** | **1.40** |
|  |  | ethanolamine | GC/MS | 1.28 | 1.06 |
|  |  | phosphoethanolamine | GC/MS | 1.03 | 0.98 |
|  |  | choline | LC/MS pos | **1.23** | 1.15 |
|  |  | glycerol 3-phosphate (G3P) | GC/MS | **0.54** | **0.36** |
|  |  | glycerophosphorylcholine (GPC) | LC/MS pos | **0.44** | **0.31** |
|  |  | cytidine 5'-diphosphocholine | LC/MS pos | 1.14 | 1.06 |
|  | Inositol metabolism | myo-inositol | GC/MS | **0.26** | **1.32** |
|  |  | inositol 1-phosphate (I1P) | GC/MS | 1.23 | 1.74 |
|  |  | scyllo-inositol | GC/MS | 1.04 | 1.19 |
|  | Lysolipid | 1-palmitoylglycerophosphoethanolamine | LC/MS pos | 0.95 | 0.87 |
|  |  | 2-palmitoylglycerophosphoethanolamine* | LC/MS pos | 0.67 | 0.64 |
|  |  | 2-palmitoleoylglycerophosphoethanolamine* | LC/MS pos | 0.49 | **0.48** |
|  |  | 1-stearoylglycerophosphoethanolamine | LC/MS neg | 1.38 | 1.14 |
|  |  | 2-oleoylglycerophosphoethanolamine* | LC/MS pos | 0.63 | **0.47** |
|  |  | 1-arachidonoylglycerophosphoethanolamine* | LC/MS neg | 1.44 | **1.81** |
|  |  | 2-arachidonoylglycerophosphoethanolamine* | LC/MS pos | 0.65 | 0.62 |
|  |  | 2-docosapentaenoylglycerophosphoethanolamine* | LC/MS pos | 0.73 | 0.67 |
|  |  | 2-docosahexaenoylglycerophosphoethanolamine* | LC/MS pos | 0.66 | **0.53** |

Heat map of statistically significant biochemicals profiled in this study. For paired comparisons, shaded cells indicate p≤0.05 (red indicates that the mean values are significantly higher for that comparison; green values significantly lower). **Blue-bolded** text indicates 0.05< p< 0.10.

**TABLE S1-6:** **KSHV infection of endothelial cells alters global host cell metabolism.**

| **SUPER PATHWAY** | **SUB PATHWAY** | **BIOCHEMICAL NAME** | **PLATFORM** | **48hpi (KSHV vs Mock)** | **96hpi (KSHV vs Mock)** |
| --- | --- | --- | --- | --- | --- |
| Lipid | Lysolipid | 1-myristoylglycerophosphocholine | LC/MS pos | 0.65 | 1.06 |
|  |  | 2-myristoylglycerophosphocholine* | LC/MS pos | 0.97 | 1.28 |
|  |  | 1-palmitoylglycerophosphocholine | LC/MS pos | 0.71 | 0.95 |
|  |  | 2-palmitoylglycerophosphocholine* | LC/MS pos | 0.55 | 0.94 |
|  |  | 1-palmitoleoylglycerophosphocholine* | LC/MS pos | **0.59** | 1.18 |
|  |  | 2-palmitoleoylglycerophosphocholine* | LC/MS pos | 0.92 | 0.91 |
|  |  | 1-stearoylglycerophosphocholine | LC/MS pos | 0.77 | 1.02 |
|  |  | 1-oleoylglycerophosphocholine | LC/MS pos | 0.73 | 1.09 |
|  |  | 2-oleoylglycerophosphocholine* | LC/MS pos | 0.70 | 0.87 |
|  |  | 2-arachidonoylglycerophosphocholine* | LC/MS pos | 0.70 | 0.84 |
|  |  | 2-docosapentaenoylglycerophosphocholine* | LC/MS pos | 0.87 | 0.86 |
|  |  | 1-stearoylglycerophosphoinositol | LC/MS neg | **2.60** | **4.94** |
|  |  | 1-arachidonoylglycerophosphoinositol* | LC/MS neg | 1.01 | **2.56** |
|  | Monoacylglycerol | 1-palmitoylglycerol (1-monopalmitin) | GC/MS | 1.05 | 1.26 |
|  |  | 1-stearoylglycerol (1-monostearin) | GC/MS | 1.13 | **1.43** |

Heat map of statistically significant biochemicals profiled in this study. For paired comparisons, shaded cells indicate p≤0.05 (red indicates that the mean values are significantly higher for that comparison; green values significantly lower). **Blue-bolded** text indicates 0.05< p< 0.10.

**TABLE S1-7:** **KSHV infection of endothelial cells alters global host cell metabolism.**

| **SUPER PATHWAY** | **SUB PATHWAY** | **BIOCHEMICAL NAME** | **PLATFORM** | **48hpi (KSHV vs Mock)** | **96hpi (KSHV vs Mock)** |
| --- | --- | --- | --- | --- | --- |
| Lipid | Diacylglycerol | 1,2-dipalmitoylglycerol | GC/MS | **0.57** | 0.99 |
|  | Sphingolipid | sphinganine | LC/MS pos | 0.95 | 1.04 |
|  |  | sphingosine | LC/MS pos | 1.01 | 1.20 |
|  | Sterol/Steroid | cholesterol | GC/MS | 0.85 | 1.00 |
|  |  | 7-dehydrocholesterol | GC/MS | **0.65** | 0.90 |
|  |  | 7-beta-hydroxycholesterol | GC/MS | 1.01 | **1.82** |
| Nucleotide | Purine metabolism, (hypo)xanthine / inosine containing | xanthine | GC/MS | 1.51 | 1.70 |
|  |  | hypoxanthine | LC/MS neg | **1.39** | **1.42** |
|  |  | inosine | LC/MS neg | 1.29 | 0.75 |
|  | Purine metabolism, adenine containing | adenine | GC/MS | 0.87 | 0.84 |
|  |  | adenosine | LC/MS neg | **0.21** | **0.24** |
|  |  | adenosine 5'-monophosphate (AMP) | LC/MS pos | 0.56 | **0.25** |
|  |  | adenosine 5'-diphosphate (ADP) | LC/MS neg | **0.40** | **0.31** |
|  | Purine metabolism, guanine containing | guanine | GC/MS | **3.02** | **4.69** |
|  |  | guanosine | LC/MS neg | 1.11 | 0.69 |
|  |  | guanosine 5'- monophosphate (GMP) | LC/MS pos | 0.69 | 0.89 |
|  | Purine metabolism, urate metabolism | allantoin | GC/MS | 0.79 | 0.99 |
|  | Pyrimidine metabolism, cytidine containing | cytidine 5'-monophosphate (5'-CMP) | LC/MS pos | **1.37** | 0.91 |
|  | Pyrimidine metabolism, orotate containing | orotate | GC/MS | 0.56 | 0.97 |
|  | Pyrimidine metabolism, uracil containing | uracil | GC/MS | **2.59** | **2.57** |
|  |  | uridine | LC/MS neg | 1.70 | **2.21** |
|  |  | uridine 5'-monophosphate (UMP) | LC/MS pos | 0.81 | 0.75 |
|  | Purine and pyrimidine metabolism | methylphosphate | GC/MS | 0.92 | 1.06 |

Heat map of statistically significant biochemicals profiled in this study. For paired comparisons, shaded cells indicate p≤0.05 (red indicates that the mean values are significantly higher for that comparison; green values significantly lower). **Blue-bolded** text indicates 0.05< p< 0.10.

**TABLE S1-8:** **KSHV infection of endothelial cells alters global host cell metabolism.**

| **SUPER PATHWAY** | **SUB PATHWAY** | **BIOCHEMICAL NAME** | **PLATFORM** | **48hpi (KSHV vs Mock)** | **96hpi (KSHV vs Mock)** |
| --- | --- | --- | --- | --- | --- |
| Cofactors and vitamins | Ascorbate and aldarate metabolism | ascorbate (Vitamin C) | GC/MS | **0.38** | **0.18** |
|  |  | threonate | GC/MS | **0.40** | **0.51** |
|  | Folate metabolism | 5-methyltetrahydrofolate (5MeTHF) | LC/MS neg | 0.88 | 0.82 |
|  | Nicotinate and nicotinamide metabolism | nicotinamide | LC/MS pos | **1.79** | 1.10 |
|  |  | nicotinamide adenine dinucleotide (NAD+) | LC/MS pos | **0.72** | 1.05 |
|  |  | nicotinamide adenine dinucleotide reduced (NADH) | LC/MS neg | **0.65** | **0.75** |
|  |  | nicotinamide adenine dinucleotide phosphate (NADP+) | LC/MS pos | 1.14 | 1.21 |
|  |  | 1-methylnicotinamide | LC/MS pos | **1.59** | **1.83** |
|  | Pantothenate and CoA metabolism | pantothenate | LC/MS pos | **0.78** | 0.94 |
|  |  | coenzyme A | LC/MS neg | 1.50 | 1.46 |
|  | Riboflavin metabolism | flavin adenine dinucleotide (FAD) | LC/MS neg | 0.98 | 1.04 |
|  | Tocopherol metabolism | alpha-tocopherol | GC/MS | 0.86 | 0.86 |
| Xenobiotics | Chemical | glycerol 2-phosphate | GC/MS | **0.65** | **0.34** |
|  |  | phenol red | LC/MS neg | **2.10** | **2.08** |
|  | Sugar, sugar substitute, starch | erythritol | GC/MS | 1.09 | 0.94 |

Heat map of statistically significant biochemicals profiled in this study. For paired comparisons, shaded cells indicate p≤0.05 (red indicates that the mean values are significantly higher for that comparison; green values significantly lower). **Blue-bolded** text indicates 0.05< p< 0.10.
